# Preference Reversals in Decision Making Under Risk are Accompanied by Changes in Attention to Different Attributes

**DOI:** 10.3389/fnins.2012.00109

**Published:** 2012-07-19

**Authors:** Betty E. Kim, Darryl Seligman, Joseph W. Kable

**Affiliations:** ^1^Department of Psychology, University of PennsylvaniaPhiladelphia, PA, USA

**Keywords:** anchoring, context effects, contingent weighting, eye-tracking, neuroeconomics, risk aversion, visual attention

## Abstract

Recent work has shown that visual fixations reflect and influence trial-to-trial variability in people’s preferences between goods. Here we extend this principle to attribute weights during decision making under risk. We measured eye movements while people chose between two risky gambles or bid on a single gamble. Consistent with previous work, we found that people exhibited systematic preference reversals between choices and bids. For two gambles matched in expected value, people systematically chose the higher probability option but provided a higher bid for the option that offered the greater amount to win. This effect was accompanied by a shift in fixations of the two attributes, with people fixating on probabilities more during choices and on amounts more during bids. Our results suggest that the construction of value during decision making under risk depends on task context partly because the task differentially directs attention at probabilities vs. amounts. Since recent work demonstrates that neural correlates of value vary with visual fixations, our results also suggest testable hypotheses regarding how task context modulates the neural computation of value to generate preference reversals.

## Introduction

A challenge for theories of decision making under risk is to account for known systematic inconsistencies in people’s decisions. An example is the “preference reversal phenomenon,” which involves systematic inconsistencies between preferences and prices (Lichtenstein and Slovic, 1971, 1973; Grether and Plott, 1979). Preference reversals were initially demonstrated by Lichtenstein and Slovic (1971). When given a choice between two gambles of similar expected value (EV), one with a high probability of winning a smaller amount of money (termed the P-bet) and another with a low probability of winning a larger amount (termed the $-bet), most people choose the higher probability P-bet. However, when providing selling prices for the same exact gambles, most people assign a higher price to the larger amount $-bet. These two decisions appear to be mutually inconsistent. The P-bet cannot be simultaneously better than *and* worse than the $-bet, and one would expect people to demand a higher price for their preferred gamble. Preference reversals violate the principle of *procedure invariance*, whereby preferences should not change depending on how they are measured (Tversky et al., 1990; Stalmeier et al., 1997).

Despite its apparent irrationality, the preference reversal phenomenon is remarkably robust. For specifically designed alternatives, the frequency of reversals can be greater than 50% (Lichtenstein and Slovic, 1973; Grether and Plott, 1979; Tversky et al., 1990). The basic inconsistency has been replicated numerous times by psychologists and experimental economists, including under different designs using non-gamble stimuli and various incentive mechanisms (Mowen and Gentry, 1980; Tversky et al., 1990; Mellers et al., 1992a,b). Further, preference reversals persist in the face of large incentives (Lichtenstein and Slovic, 1973; Grether and Plott, 1979), including when the experimenter exploits the inconsistency to take money from the subject (Berg et al., 1985; Chu and Chu, 1990).

Various explanations have been proposed for preference reversals, which attribute the reversal to changes at different stages of the decision process. Different theories attribute preference reversals to changes in how attributes are weighted (Tversky et al., 1988), changes in how weighted attributes are combined to form an evaluation (e.g., additive vs. multiplicative combination; Mellers et al., 1992b), or changes in how a formed evaluation is expressed, or translated into a response, in different tasks (Goldstein and Einhorn, 1987). Though conceptually distinct, changes at these different stages are also not mutually exclusive.

A prominent explanation for preference reversals is Tversky et al. (1988) *contingent weighting* hypothesis. They argue that attribute weights are closer to lexicographic (i.e., closer to all-or-none) in choice compared to other tasks, which leads to the most important attribute being weighted even more heavily in choice, a phenomena called the *prominence effect* (Slovic, 1975; Tversky et al., 1988). Since most people are risk-averse (Holt and Laury, 2002), weighting probability more than amount, this would lead to the probability dimension being weighted even more in choice than other decision tasks (Note there is some debate, though, about whether the prominence effect occurs for gambles; see Tversky et al., 1988, p. 382). By contrast, Tversky et al. (1988) argue that the payoff dimension is weighted more during bids because of the *compatibility effect*, whereby attributes that are compatible with the output are given more weight (in this case, payoff is compatible with bids, since both are in dollars; Slovic, 1975; Tversky et al., 1988). Formally, Tversky et al. (1988) model the change in responses across the two tasks as a change in the weight α*_i_* (where *i* = choice, bid) of the following utility function for a gamble to win amount *a* with probability *p*:

$$U\left(p,a\right)=\log p+\alpha_{i}\log a$$

Note that this is simply the logarithmic transform of an expected utility (EU) model in which the degree of risk aversion varies between choices and bids.

Here, using visual fixations as an index of information processing and visual attention, we sought to determine what information people attend to during a preference reversal paradigm. Specifically, we aimed to test whether visual fixations reflect changes in the weighting of different attributes, with people looking at probability information more during choices and amount information more during bids. Since preference reversals could be due to changes at different stages of the decision process, this finding would also provide additional support for contingent weighting being at least part of the explanation.

This experiment also builds on recent research linking visual fixations and preferences. Rangel and colleagues have shown that visual fixations both reflect and influence preferences between goods (Armel et al., 2008; Krajbich et al., 2009, 2010). Visual fixations also modulate the neural correlates of preferences, with activity in ventromedial prefrontal cortex and ventral striatum reflecting the value of the fixated item compared to the value of item not fixated (Lim et al., 2011). Here we test whether the link between fixations and preferences generalizes to decision making under risk, and whether fixations are further linked to attribute weights. Given the link between fixations and neural correlates of preferences, this evidence should also inform theorizing regarding the specific neural signals that might be modulated by task context to give rise to preference reversals.

Our investigation follows previous process tracing studies by Johnson et al. (1988) and Schkade and Johnson (1988). Using Mouselab, they found that individuals spent proportionally more time looking at probability information during choices than during bidding. However, Mouselab may not always provide the most natural decision environment (Lohse and Johnson, 1996). In Mouselab, subjects acquire information by positioning a mouse cursor over different windows, and the pattern of mouse movements is recorded. This can increase the amount of effort needed to acquire information, which can then alter the information processing behavior of subjects (Lohse and Johnson, 1996). Eye-tracking does not have this problem. Since eye-tracking does not impose additional requirements on subjects to obtain or maintain information, it might in some cases provide a more sensitive or more accurate measure of information processing. For this reason, as well as to build on recent work linking visual fixations and preferences, we thought it was important to further investigate preference reversals using eye-tracking techniques.

## Materials and Methods

### Participants

Twenty-six paid volunteers from the University of Pennsylvania community participated in this study. Data from two participants were discarded because their responses suggested confusion regarding the bidding task. One participant’s bids were not positively correlated with EV, and the other participant bid higher than the amount to win in several gambles. The mean age of our final sample (*N* = 24) was 23.6 years (age range: 19–29 years), and 52% were female. All participants gave written informed consent in accordance with the procedures of the Institutional Review Board at the University of Pennsylvania.

### Tasks and stimuli

**Figure 1:**
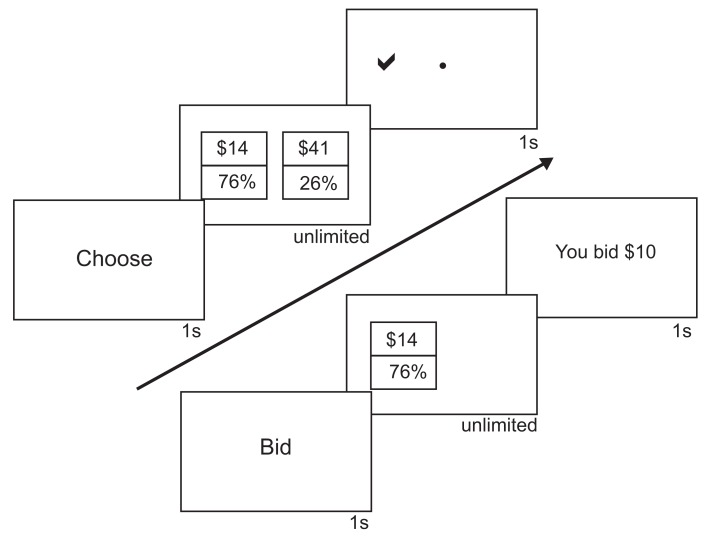
**Choice and bid tasks**. The sequences of events within a trial for both choice and bid trials are shown. For choice trials, subjects saw “Choose” for 1 s. Subjects then saw two gambles, a $-bet gamble and a P-bet gamble. Subjects had unlimited time to choose one of the gambles. After submitting their response, subjects would see a check mark on the side of the chosen gamble. For bid trials, subjects saw “Bid” for 1 s. Subjects then saw one gamble, either a $-bet or a P-bet gamble, on the left side of the screen. To the right of the gamble was a “$” where subjects bids would appear. Subjects had unlimited time to submit their bids. After submitting their response, subjects would see the amount they bid.

On each trial, subjects either made a choice between two gambles (choice trials) or provided their evaluation of a single gamble (bid trials, see Figure 1). On choice trials, subjects chose between two different gambles with varying probabilities (12–95%) of winning different amounts of money ($10–$98). On bid trials, subjects entered their subjective evaluation of a gamble in dollar amounts. At the end of each session, one trial was randomly selected, and participants were paid according to their decision on that trial. If subjects won money, they received that money in addition to the show-up fee of $10.

We used E-Prime to present all behavioral stimuli (Psychology Software Tools, Pittsburgh, PA, USA). Subjects entered their responses using a keyboard. Subjects were presented with a total of 100 bid trials and 100 choice trials in eight alternating blocks of 25 trials each. In case placement of the probabilities and amounts biased decision making, half the subjects saw the amounts as the top number and the other half saw the probabilities as the top number. All subjects saw the same set of gambles in the same order. During a choice trial, subjects were presented with a screen with the word “Choose” for one second. They then saw a screen with two gambles side-by-side and had unlimited time to choose between the two gambles. Subjects pressed “1” to choose the gamble on the left side of the screen and pressed “0” to choose the gamble on the right. During a bid trial, subjects were presented with a screen with the word “Bid” for 1 s. They then saw a screen with a single gamble and had unlimited time to enter their dollar bid. Subjects used the number keys to enter their bid and submitted their response by pressing the “return” key. Once bids were entered, subjects were unable to change their responses. Participants were instructed to bid the “smallest amount of money (they) would be willing to exchange for the opportunity to play the gamble.”

Subjects went through a training period in the beginning to ensure understanding of the task. Subjects had two practice trials for each of the trial types. On bid practice trials, subjects were taken through a series of questions after they entered their bid. These questions were used during training to ensure that subjects understood the bidding task and could provide well-calibrated bids. First, subjects were asked if they would forego playing out the gamble to take a counteroffer that was $1 higher than their bid. If they answered “no,” they were told they bid too low and were asked to bid again. If subjects answered “yes,” they were then asked if they would play out the gamble and forego taking a counteroffer $1 less than their bid. If they answered “no,” they were told they bid too high and were asked to bid again. Subjects repeated this process until they answered yes to both questions. These questions were only asked on practice trials, and were not included on experimental trials.

In choice trials, one gamble had a high probability of winning a small amount of money (termed the P-bet, e.g., 84% chance of $20), and the other had a low probability of winning a larger amount (termed the $-bet, e.g., 24% chance of $70). Fifty pairs of P-bets (ranging from 70 to 95% chance of winning $10–$34) and $-bets (ranging from 12 to 37% chance of winning $35–$98) were selected so that the P-bet and $-bet were approximately equal in EV, with differences ranging from $0.00 to $0.09 and a median difference of $0.02. Probability ranges were chosen based on previous studies (e.g., Lichtenstein and Slovic, 1971) and ensured the ranges for P-bets and $-bets did not overlap. Amounts were chosen to provide a reasonable range of EV, given that subjects would be paid according to the outcome on a single trial. No probability or dollar amount was used more than twice in the stimulus set. This stimulus set was pre-tested in pilot behavioral subjects (*n* = 12) who demonstrated a robust preference reversal effect, and has now been used in several studies in our laboratory. To encourage participants to attend to each choice and avoid following a simple heuristic (such as always choosing the higher probability gamble), 10 of the 50 pairs were mismatched so that either the P-bet or $-bet had a much higher EV. The EV across all gamble pairs varied from $8.10 to $29.23, with a median of $18.13. Each pair was presented twice during choice trials, with the left-right placement of the gambles switching between presentations. The same gambles used in the choice task were shown once individually in the bidding task. Thus for each subject we have 100 choice and 100 bid trials where the stimulus on the left of the screen is identical, and what differs is the presence of another gamble or the bid prompt on the right side of the screen.

Both tasks were administered in an incentive-compatible manner. At the end of the experiment, participants rolled dice to randomly determine one bid or choice trial to be played out for real money. If a choice trial was selected, participants were given the opportunity to play the gamble that they chose, using a 100-sided die to determine the outcome. For example, if the chosen gamble was a 75% chance of winning $21, a roll of 75 or below on the die would pay $21 and a roll of 76 or above would pay $0. If a bid trial was selected, participants were paid using the Becker–DeGroot–Marschak (BDM) method, a widely used incentive-compatible procedure (Becker et al., 1964). The subject’s bid on the selected gamble was compared to a randomly generated counteroffer (between $0 and the amount to win), created by dividing the roll of a 100-sided die by 100 and multiplying the resulting fraction by the amount to win. If the subject’s bid was higher than the counteroffer, the subject played the gamble. If the subject’s bid was lower than the counteroffer, the subject received the counteroffer amount. This method incentivizes participants to bid their true valuation of the gamble, the amount at which they would be indifferent between receiving their bid and playing the gamble. The amount of money subjects won varied from $0 to $37.41 with a median of $21.

### Eye-tracking

We used an Eyelink II head-mounted eye-tracker (SR Research Ltd., Mississauga, ON, Canada) to monitor participant’s eye movements during the task. A camera imaged the participant’s right eye at 250 Hz. Subjects sat approximately 18′′ from the screen and were calibrated using a 9-point calibration. To manage eye drift and head movement, the subject fixated on a black dot at the center of the screen after each trial and a drift correction measured how much each subject’s measured gaze differed from the center of the screen. The experimenter monitored drift corrections throughout the whole experimental session and re-calibrated when the subject’s gaze drifted from the center. Eye movements were recorded during each trial between the time of the first stimuli and the time of the subject’s response.

### Behavioral analysis

We used Matlab (Mathworks, Natick, MA, USA) and SPSS (SPSS Inc., Chicago, IL, USA) to analyze our behavioral and eye-tracking data. For each pair of gambles, we categorized responses in the choice task according to whether the subjects chose the P-bet both times (“chose P”), chose each bet once (“chose=”), or chose the $-bet both times (“chose $”). Participants were consistent about 79% of the time, choosing the same gamble across both choices. In the bid task, we categorized responses according to whether the subject bid higher on the P-bet (“bid P”), bid equal amounts for both bets (“bid=”), or bid higher on the $-bet (“bid $”). Within the 40 gamble pairs matched in EV, we calculated two measures of the preference reversal effect. One measure included all instances of increasing preference for the $-bet (“weak P-to-$ reversals”), that is, when subjects chose the P-bet both times then bid equal amounts, when they chose each bet once then bid higher on the $-bet, or when they chose the P-bet both times then bid higher on the $-bet. The other measure included only this last category, instances where the subject chose the P-bet twice and then bid higher on the $-bet (“strict P-to-$ reversals”). We also calculated two similar measures for reversals in the unpredicted direction, from the $-bet in choice to the P-bet in bids.

In addition, we estimated a model in both tasks that assumed subjects’ decisions were a function of the EU of the gambles:

$$\text{EU}\left(p,a\right)=p\times a^{\alpha_{\text{i}}}$$

Here α*_i_* (where *i* = choice, bid) is a measure of risk aversion. An α*_i_* equal to one leads to risk-neutral decisions, an α*_i_* less than one to risk-averse decisions, and an α*_i_* greater than one to risk-seeking decisions. As mentioned in the introduction, one simple model of contingent weighting is merely the logarithmic transform of this equation (Tversky et al., 1988). From that perspective, an α*_i_* equal to one means equal weighting, an α*_i_* less than one means probability is weighted more strongly, and an α*_i_* greater than one means amount to win is weighted more strongly.

For choices, we fit a logistic regression that assumed choice probabilities (cp) were a function of the difference in expected utility between the two gambles:

$$\text{cp}\left(\text{E}{\text{U}}_{1},\text{E}{\text{U}}_{2}\right)=\frac{1}{1+{\text{e}}_{}^{\beta \left(\text{E}{\text{U}}_{1}-\text{E}{\text{U}}_{2}\right)}}$$

We fit this equation for each subject to his/her observed choices using an iterative optimization in MATLAB (fminsearch and fminunc) to find the maximum likelihood estimate of α_choice_ and β. The α_choice_’s of two subjects exceeded the boundaries that our model could reliably estimate (0.17 < α_choice_ < 5.05), so we excluded both α’s from these subjects from further analysis. For bids, we fit a model that assumed the subject’s bid was equal to the expected utility of the gamble, using non-linear least squares in MATLAB. We obtained almost identical results to those reported below if we fit α_choice_ and α_bid_ using the logarithmic transform of expected utility (i.e., the contingent weighting equation in the introduction).

Response time was calculated as starting from the onset of the stimuli and ending when the participant submitted their responses.

Placement of the amounts and probabilities did not have any significant effects on choice and bidding behavior (i.e., strict or weak P-to-$ reversals, α_choice_ or α_bid_). All *p*s > 0.10.

### Eye tracking analysis

We used Data Viewer (SR Research Ltd., Mississauga, ON, Canada) for all pre-processing of the eye-tracking data and Matlab (Mathworks, Natick, MA, USA) for all eye-tracking analysis. The Eyelink II software automatically parses eye movement data into fixations, blinks, and saccades based on standard saccade thresholds (velocity threshold = 30°/s, acceleration threshold = 8000°/s^2^). Only fixations initiated after the onset of the gambles were included in our analyses. Additionally, the Eyelink on-line parser denoted a blink when the pupil was very small, or when the eye-camera image of the pupil was missing or severely distorted by eyelid occlusion.

We defined regions of interest (ROI) corresponding to each amount and probability within each trial. The size of the screen was 800 by 1200 pixels, and each ROI was approximately 280 by 320 pixels. There were four ROIs in choice trials, and two ROIs during bid trials. For a controlled comparison between choice and bid trials, we focused our analyses on only the two ROIs for the left gamble in choice trials, since these were visually identical to and contained the same amount of physical space as the two ROIs in bid trials. For fixations and looking durations (but not first fixations), we observed the same pattern of results if we collapsed across all four ROIs in choice trials.

We included three dependent variables in our eye-tracking analyses: number of fixations, looking duration, and the first fixation of each trial. For each of our dependent variables, we ran an ANOVA with gamble type (P-bets vs. $-bets), attribute (probability vs. amount), and trial type (choice vs. bids) as within-subject factors and attribute placement (probability on top vs. amount on top) as a between-subject factor. We refer to this ANOVA below as our between-task analysis. To test subsequent comparisons within a trial type, we ran separate ANOVAs for choice trials and bid trials with gamble type (P-bets versus $-bets) and attribute (probability vs. amount) as within-subject factors and attribute placement (probability on top vs. amount on top) as a between-subject factor. We refer to these ANOVAs below as within-task analyses. These analyses were all done using raw fixation numbers and looking times, but we observed the same pattern of results if we examined ratios of these variables (e.g., the ratio of fixations on probability versus amount, etc.). Fixations and looking durations for gamble types and attribute were highly correlated. All *r*s > 0.92, *p*s < 001.

For fixations and looking durations (but not first fixations), placement of the amounts and probabilities did not interact with the eye-tracking effects reported below. There was, however, an interaction between attribute and attribute placement for all three dependent measures. Subjects had more total fixations [mean = 5.69 ± 0.46 fixations vs. mean 4.77 ± 0.48 fixations; *F*(1, 22) = 33.37, *p* < 0.001], longer looking durations [mean = 1,718 ± 208 ms vs. mean = 1,337 ± 192 ms; *F*(1, 22) = 22.15, *p* < 0.001], and more first fixations [mean = 77 ± 3% vs. mean = 23 ± 3%; *F*(1, 22) = 87.54, *p* < 0.001] for the attribute that was presented on top.

Finally, to test for any effects of individual differences, we looked at the correlation between each of our eye-tracking dependent variables (proportion of total fixations and looking duration by trial type and gamble type; proportion of total fixations, looking duration, and first fixations by trial type and attribute) and each of our behavioral variables (number of strict and weak P-to-$ reversals, α_choice_ and α_bid_). This analysis excluded the two subjects whose choice alphas exceeded the boundaries that we could reliably estimate (these two subjects were also outliers in terms of the number of reversals, with neither making any weak P-to-$ reversals while the minimum among the remaining subjects was 22 weak reversals).

## Results

### Behavioral results

Overall, subjects spent more time on bid trials than on choice trials. There was a significant increase in response times from choice trials to bid trials, *F*(1, 23) = 74.95, *p* < 0.001. The average response time was 4,257 ± 549 ms during choice trials and 6,894 ± 485 ms during bid trials. [Note that, presumably secondary to this reaction time effect, there were also more total fixations, *F*(1, 22) = 43.57, *p* < 0.001, and longer looking durations, *F*(1, 22) = 40.45, *p* < 0.001, during bid trials than during choice trials.] Within bid trials, subjects took longer to bid on $-bets than on P-bets, *F*(1, 23) = 31.43, *p* < 0.001. The average response time for bids on P-bets was 6,381 ± 97 ms and the average response time for $-bets 7,394 ± 101 ms.

**Figure 2:**
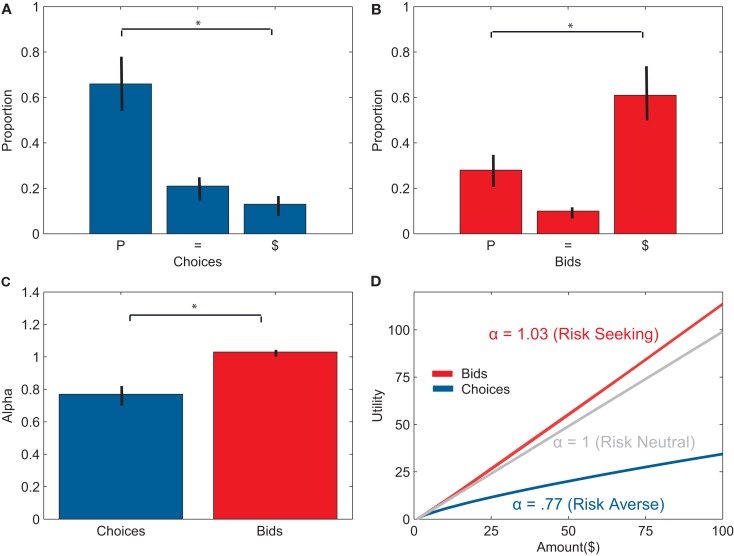
**(A)** Percentage of gamble pairs where subjects chose the P-bet option twice (P), the $-bet twice ($), or both equally (=). On average, subjects chose the P-bet significantly more than the $-bet. **(B)** Percentage of gamble pairs where subjects bid higher for the P-bet option (P), $-bet option ($), or bid the same amount for both gambles (=). On average, subjects bid higher on $-bets than on the P-bets**. (C)** Average alpha values for choice trials and bid trials. Alphas were significantly higher for bidding than for choice. **(D)** The average expected utility function for bids and choices given the inferred alphas. Subjects were risk-averse during choices and slightly risk-seeking during bids.

Subjects also demonstrated a robust preference reversal effect. During choice trials, subjects chose the P-bet significantly more often than the $-bet, *F*(1, 23) = 34.02, *p* < 0.001. On average, subjects chose the P-bet both times for 66 ± 13% of the pairs, chose equally for 21 ± 4% of the pairs and chose the $-bet both times for 13 ± 3% of the pairs (see Figure 2A). In contrast, subjects bid significantly higher on the $-bet than on the P-bet, *F*(1, 23) = 18.22, *p* < 0.001. Subjects bid higher on the $-bet for 61 ± 13% of the pairs, bid the same on both gambles for 10 ± 2% of the pairs, and bid higher on the P-bet for 28 ± 6% of the pairs (See Figure 2B). Subjects preferred the P-bet significantly more often when choosing than when bidding, *F*(1, 23) = 40.54, *p* < 0.001, and preferred the $-bet significantly less often when choosing than when bidding, *F*(1, 23) = 49.21, *p* < 0.001.

Across all gamble pairs, subjects exhibited increased preference for the $-bet in bids more often than the reverse effect, *F*(1, 23) = 104.37, *p* < 0.001. Subjects made weak P-to-$ reversals for 67 ± 5% of gamble pairs and weak $-to-P reversals for 10 ± 3% of gamble pairs. Subjects also exhibited significantly more strict P-to-$ reversals, choosing the P-bet both times and bidding higher on the $-bet, than strict $-to-P reversals, choosing the $-bet both times and bidding higher on the P-bet, *F*(1, 23) = 53.30, *p* < 0.001. Subjects made strict P-to-$ reversals for 37 ± 4% of gamble pairs and strict $-to-P reversals for less than 1 ± 1% of gamble pairs. For pairs where the subject chose the P-bet both times, they bid an average of $10.37 ± 2.35 higher on $-bet.

Preference reversals were also evident by changes in risk aversion, or attribute weighting, in the two tasks. Subjects were risk-averse, weighting probability more, during choice trials (α_choice_ = 0.77, SE = ±0.05). In contrast, subjects were close to risk-neutral, weighting probability and amount almost equally during bid trials (α_bid_ = 1.03, SE = ± 0.01; see Figures 2C,D). α_choice_’s were significantly smaller than α_bid_’s, *t*(21) = −4.37, *p* < 0.001.

### Eye-tracking results

For eye-tracking analyses, our main dependent variables were number of fixations and looking durations. Both of these variables showed strong effects of task context. In each task, subjects looked more at the preferred gamble type (P-bet in choices, $-bet in bids) and the more heavily weighted attribute (probability in choices, amount to win in bids).

**Figure 3:**
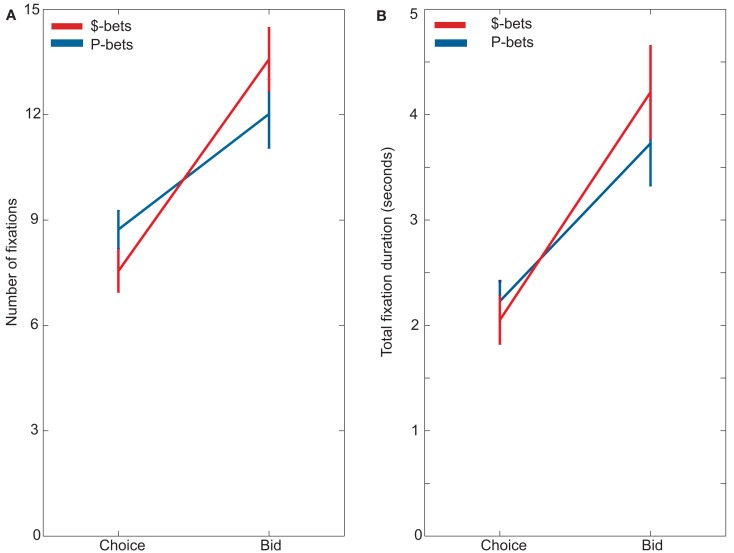
**(A)** Average number of fixations of $-bets and P-bets during choices and bids. **(B)** Average duration looking at $-bets and P-bets during choices and bids.

Subjects looked at the preferred gamble type more, fixating on P-bets more often during choice trials and $-bets more often during bid trials (Figure 3). This was evidenced by a significant interaction between trial type and gamble type for both the number of fixations, *F*(1, 22) = 44.25, *p* < 0.001, and for the duration of fixations, *F*(1, 22) = 23.53, *p* < 0.001, in our between-task analysis. Looking within each task, subjects made significantly more fixations on P-bets (mean = 8.73 ± 0.55) than on $-bets (mean = 7.55 ± 0.61) during choice trials, *F*(1, 22) = 27.48, *p* < 0.001. Subjects also spent significantly more time looking at P-bets (mean = 2,229 ± 191 ms) than at $-bets (mean = 2,050 ± 231 ms) during choice trials, *F*(1, 22) = 7.77, *p* = 0.01. In contrast, during bid trials, subjects made more fixations on $-bets (mean = 13.60 ± 0.98) than on P-bets (mean = 12.05 ± 0.93; *F*(1, 22) = 22.75, *p* < 0.001) and spent more time looking at $-bets (mean = 4,213 ± 445 ms) than at P-bets (mean = 3,727 ± 405 ms; *F*(1, 22) = 16.68, *p* < 0.001).

**Figure 4:**
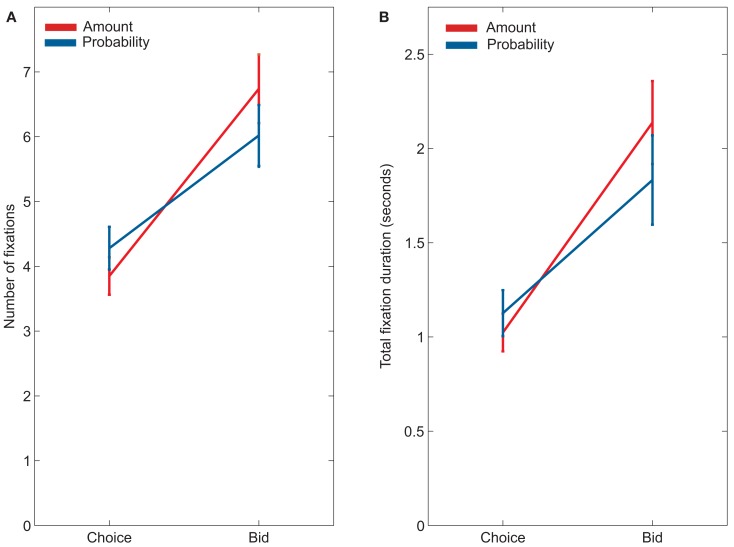
**(A)** Average number of fixations of probabilities and amounts during choices and bids. **(B)** Average duration looking at probabilities and amounts during choices and bids.

Fixations of the two attributes, probability and amount, also differed between choice and bid trials. Subjects were more likely to look at probabilities during choice and more likely to look at amounts during bidding (Figure 4). This was evidenced by a significant attribute by trial type interaction for both number of fixations, *F*(1, 22) = 14.13, *p* < 0.01, and looking durations, *F*(1, 22) = 4.29, *p* < 0.05, in our between-task analysis. Looking within each task, subjects made significantly more fixations on probability (mean = 4.3 ± 0.32 fixations) than on amount (mean = 3.9 ± 0.30 fixations) during choice trials, *F*(1, 22) = 5.57, *p* < 0.05. Similarly, subjects spent marginally more time looking at probability (mean = 1,126 ± 122 ms) than at amount (mean = 1,012 ± 100 ms) during choice trials, *F*(1, 22) = 4.19, *p* = 0.05 [this effect was more reliable when considering both gambles, instead of just the left gamble: duration on probability = 2,121 ± 233 ms, duration on amount = 1,865 ± 191 ms, *F*(1, 22) = 5.99, *p* < 0.05]. In contrast, during bid trials, subjects made significantly more fixations on amount (mean = 6.74 ± 0.53 fixations) than on probability [mean = 6.06 ± 0.47 fixations; *F*(1, 22) = 8.12, *p* < 0.01], and spent marginally more time looking at amount (mean = 2,137 ± 220 ms) than at probability [mean = 1,832 ± 237 ms; *F*(1, 22) = 2.97 *p* < 0.10].

There was further interaction between these effects of gamble type and attribute. Specifically, the interaction between trial type and attribute was greater for $-bets than for P-bets. This was evidenced by a significant three-way interaction between trial type, gamble type, and attribute for both fixations, *F*(1, 22) = 5.43, *p* < 0.05, and for looking duration, *F*(1, 22) = 11.32, *p* < 0.01, in our between-task analysis.

We also examined which attribute was fixated on first in choice and bid trials. First fixations were more likely to be on probability than on amount across both kinds of trials (mean first fixation on probability = 59 ± 13%; *F*(1, 22) = 9.70, *p* < 0.01 in our between-task analysis). Looking within each task, probability was more likely to be fixated on first in both choice trials, *F*(1, 22) = 11.06, *p* < 0.01, and in bid trials, *F*(1, 22) = 4.62, *p* < 0.05. This was qualified by a significant interaction between attribute and trial type [*F*(1, 22) = 5.88, *p* < 0.05], with probability more likely to be fixated on first in choice trials (62 ± 5% in choice trials vs. 55 ± 8% in bid trials). This interaction, however, was not reliable when we included both choice options (all four ROIs) in the analysis, rather than restricting our analysis to only the left choice option [*F*(1, 22) = 1.27, *p* > 0.10]. The two-way interaction between attribute and trial type was further qualified by a three-way interaction between attribute, trial type, and attribute order, *F*(1, 22) = 52.32, *p* < 0.001, in our between-task analysis. This interaction arose because during bid trials, subjects primarily fixated on the top attribute first (mean = 88 ± 3% of first fixations), regardless of whether it was probability or amount. Subjects fixated on the top attribute first to a lesser degree during choice trials (67 ± 5% of first fixations). Thus it appears attribute placement had a stronger effect on first fixations than attribute identity.

Finally, we tested for any effects of individual differences by examining the correlations between the eye-tracking measures and behavioral measures. Only two of these correlations were statistically significant. Individuals who fixated on the P-bet more during choice (evaluated using either fixations or looking duration) were more risk-averse, *r*s = −0.66 and −0.61, *p*s < 0.01, respectively. Note these correlations remained significant even when using a Bonferroni correction for the number of correlations examined.

## Discussion

Here we replicated the preference reversal phenomenon in decision making under risk, in which people facing two gambles of equal EV choose the one with the higher probability of winning, but assign a higher price to the one with the larger potential payoff. We have additionally shown that preference reversals are accompanied by changes in visual fixations. Participants had more fixations on the preferred gamble in each task (P-bets in choices, $-bets in bids). They also had more fixations on the more heavily weighted attribute in each task (probability in choices, amounts in bids). These results show that visual fixations reflect preferences in decision making under risk, as they do in decisions about goods (Krajbich et al., 2009, 2010), and that fixations further reflect attribute weights in a multi-attribute choice paradigm. These results support a contingent weighting explanation of preference reversals, and also suggest testable hypotheses about the neural mechanisms of preference reversals.

Behaviorally, we replicated the classic preference reversal finding. Our participants predominantly chose the high-probability bet from a pair of gambles matched in EV, and predominantly assigned higher prices to the (alternative) bet that offered the larger amount to win. For 37% of gamble pairs, our participants made strict P-to-$ reversals, choosing the P-bet twice and bidding higher on the $-bet. Consistent with this, participants were overall risk-averse during choices, and very slightly risk-seeking during bids.

One novel aspect of our paradigm compared to previous work is the highly repeated nature of the trials. Participants made 100 choices and 100 bids over the course of the experiment. Our results demonstrate that preference reversals are not eliminated when subjects are tested with many repeated trials. Our design does not allow us to test whether they are diminished by repeated trials, though the effects we observed in this experiment are of similar size to those reported in the literature. Most neuroscientific methods require many repeated trials and within-subject comparisons. While many context effects are eliminated under these conditions, our results show that preference reversals are not, and therefore may be a good paradigm for neuroscientific studies of context effects.

Despite only having to assess the value of one gamble, participants took longer to make bids than to make choices. Although it is possible that the difference in response times might be due to differences in response entry, it is unlikely that pressing one or two more buttons accounts for an increase of more than 2 s. Spending more time deciding on a bid than choosing between two options is consistent with previous findings (Johnson et al., 1988; Schkade and Johnson, 1988). It suggests that the decision process for assigning prices is potentially more complex than that required for binary choices. This is consistent with models that assume that binary choice is the more basic process (Johnson and Busemeyer, 2005), but not with models that assume pricing is more basic (Luce et al., 1993). Pricing and matching tasks have rarely been studied in decision neuroscience (though see Plassmann et al., 2007) so an interesting question for future research is the degree to which choice and bidding rely on shared vs. distinct neural processes.

Recent work has found that fixations reflect trial-to-trial variability in preferences (Krajbich et al., 2009, 2010). Our findings extend this principle to decision making under risk. During choices, participants made more fixations on the preferred gamble type in that task, P-bets, and spent a greater amount of time looking at P-bets. During bids, participants made more fixations on the preferred gamble type in that task, $-bets, and spent a greater amount of time looking at $-bets. We acknowledge that the bidding results are confounded by a longer reaction time for $-bets than for P-bets, making this finding more difficult to interpret. There is not such a confound in the choice results, however, which clearly replicate the link between fixations and preferences observed in other choice domains.

Our key finding, though, was that preference reversals were associated with changes in visual fixations to the two gamble attributes in the two tasks. During bidding, participants made more fixations on amounts and spent a greater amount of time looking at amounts. During choices, participants made a greater number of fixations on probabilities and spent a greater amount of time looking at probabilities.

The directionality of these results is broadly consistent with the contingent weighting hypothesis. According to this hypothesis, preference reversals result from an increased weight on probability in value computations during choice, and a corresponding increased weight on amount during bids. We found that people fixate probabilities more during choice and amounts more during bids.

These differences in fixations might only be an index of the differential weighting of attributes, or alternatively might also be a cause of this differential weighting. This latter possibility raises several ideas for future research that would involve exogenously controlling fixations. If fixations influence attribute weighting, then preference reversals might be reduced, or even eliminated, when participants are forced to look equally at probabilities and amounts. In addition, forcing more fixations to the weaker attribute of an option might make people less likely to choose that option, a potential exception to previous work showing that fixating on an option makes people more likely to choose it (Armel et al., 2008).

However, a simple model in which preference reversals are due solely to changes in attribute weights, and fixations provide an unbiased index of these weights, has trouble completely accounting for our data. As shown in Figure 2D, participants’ decisions reflect nearly equal weighting of probability and amount during bids (i.e., participants are close to risk-neutral), and a greater weighting of probability during choices (i.e., participants are risk-averse). In contrast, as shown in Figure 4, participants fixate probabilities more during choices and amounts more during bids.

One possible resolution is that people are intrinsically risk-averse, weighting probabilities more, and only changes from that intrinsic baseline are reflected in changes from equal fixation of the two attributes. Another possibility is that fixations are monotonically, but not linearly, related to attribute weights. While participants are close to risk-neutral during bids, they are still significantly risk-seeking, and they also fixate amounts more than probabilities. A final possibility, of course, is that fixations and looking times reflect more than attribute weights alone. For example, first fixations showed a strong effect of the spatial position of attributes, and other influences could have shifted fixations similarly in both choices and bids.

Our findings are similar to those reported previously by Johnson et al. (1988) and Lohse and Johnson (1996). Using Mouselab, those authors found that subjects attended to amounts more, and probabilities less, during bids than during choices (for example, 56 vs. 51% of the time in Experiment 1 of Schkade and Johnson, 1988). This same overall pattern was arguably more dramatic in our fixation data. This points to a potential difference in sensitivity between the two techniques, which might arise from how people process information differently in the two environments. In the Mouselab environment, only one piece of information is available at any one time. Johnson et al. noted that in their experiments some subjects used a strategy of first looking at all of the information sequentially, and then holding it in mind while they made their decision. Under free viewing, subjects do not adopt this strategy at all. Of the total fixations in Figure 3, 3.59 ± 0.28 fixations during choice trials are made when returning to an item after fixating on it once and then looking elsewhere, while 5.40 ± 0.42 represent return fixations during bidding.

Our data on individual differences provide additional support for the notion that fixations reflect preferences during choices. Individuals who fixated more on the P-bet during choice trials were more risk-averse. However, we did not find any other significant correlations between individual differences in eye movements and behavioral measures. A possible reason for these null findings is that we have a small sample size for evaluating individual differences. Additionally, most participants show a robust preference reversal effect, so there is limited variability in the number of preference reversals. Future research could further explore how individual differences in fixations related to individual differences in preference reversals, perhaps using a larger sample or a paradigm in which there is greater variance in the behavioral effect.

Future research could also investigate how different presentation formats affect eye fixations and, in turn, preference reversals. For example, Johnson et al. (1988) have shown that different presentation formats can move around preference reversals and that these changes are associated with changes in information processing. Specifically, when probabilities are more complex (e.g., 399/456) the number of preference reversals increases. In addition, subjects spent a greater proportion of time viewing probability information when probabilities were displayed as complicated fractions, and subjects who spent more time on probability also demonstrated more reversals. We do not know of any similar studies looking at the relationship between visual fixations and decisions under risk when presentation format varies, though this would be an interesting follow-up to our study.

Another interesting question for future research concerns the neural mechanism of preference reversals. Several studies have now demonstrated that BOLD activity in ventromedial prefrontal cortex and ventral striatum is correlated with the subjective value of the options under consideration during decision making (Kable and Glimcher, 2009). A recent study showed that value-related activity in these regions is further modulated by visual fixations, tracking the value of the fixated item compared to the item not fixated (Lim et al., 2011). Paired with our findings, this suggests the intriguing hypothesis that BOLD activity in ventromedial prefrontal cortex and ventral striatum differentially reflects probabilities and amounts during choices and bids. That is, in a preference reversal paradigm, BOLD activity in these regions might be more strongly affected by probabilities during choice and more strongly affected by amounts during bids. Such a finding would also suggest that neural correlates of probability and magnitude (Knutson et al., 2005) could depend on the task context.

In conclusion, we found that preference reversals in decision making under risk were accompanied by differential attention to probabilities vs. amounts. The directionality of this effect was consistent with a contingent weighting explanation (Tversky et al., 1988), with people looking at probabilities more during choice and amounts more during bids. Given recent work demonstrating neural correlates of value (Kable and Glimcher, 2009), which are modulated by visual attention (Lim et al., 2011), this work suggests testable hypotheses regarding how task-dependent strategies might alter the weighting of attributes in the neural computation of value to cause preference reversals.

## Conflict of Interest Statement

The authors declare that the research was conducted in the absence of any commercial or financial relationships that could be construed as a potential conflict of interest.
